# Attention-guided deep learning for gestational age prediction using fetal brain MRI

**DOI:** 10.1038/s41598-022-05468-5

**Published:** 2022-01-26

**Authors:** Liyue Shen, Jimmy Zheng, Edward H. Lee, Katie Shpanskaya, Emily S. McKenna, Mahesh G. Atluri, Dinko Plasto, Courtney Mitchell, Lillian M. Lai, Carolina V. Guimaraes, Hisham Dahmoush, Jane Chueh, Safwan S. Halabi, John M. Pauly, Lei Xing, Quin Lu, Ozgur Oztekin, Beth M. Kline-Fath, Kristen W. Yeom

**Affiliations:** 1 Department of Electrical Engineering, Stanford University, Stanford, CA USA; 2 Stanford University School of Medicine, Stanford, CA USA; 3 Department of Radiology, Lucile Packard Children’s Hospital, Stanford University School of Medicine, Stanford, CA USA; 4 Department of Radiology, St. Joseph’s Hospital and Medical Center, Phoenix, AZ USA; 5 Department of Radiology, Children’s Hospital Los Angeles, Los Angeles, CA USA; 6 Department of Obstetrics and Gynecology, Lucile Packard Children’s Hospital, Stanford University School of Medicine, Stanford, CA USA; 7 Department of Radiation Oncology, Stanford University School of Medicine, Stanford, CA USA; 8 Philips Healthcare North America, Gainesville, USA; 9 Department of Neuroradiology, Bakırçay University, Çiğli Education and Research Hospital, İzmir, Turkey; 10 Department of Radiology, Cincinnati Children’s Hospital Medical Center, University of Cincinnati College of Medicine, Cincinnati, OH USA

**Keywords:** Machine learning, Paediatric research, Brain imaging

## Abstract

Magnetic resonance imaging offers unrivaled visualization of the fetal brain, forming the basis for establishing age-specific morphologic milestones. However, gauging age-appropriate neural development remains a difficult task due to the constantly changing appearance of the fetal brain, variable image quality, and frequent motion artifacts. Here we present an end-to-end, attention-guided deep learning model that predicts gestational age with R^2^ score of 0.945, mean absolute error of 6.7 days, and concordance correlation coefficient of 0.970. The convolutional neural network was trained on a heterogeneous dataset of 741 developmentally normal fetal brain images ranging from 19 to 39 weeks in gestational age. We also demonstrate model performance and generalizability using independent datasets from four academic institutions across the U.S. and Turkey with R^2^ scores of 0.81–0.90 after minimal fine-tuning. The proposed regression algorithm provides an automated machine-enabled tool with the potential to better characterize in utero neurodevelopment and guide real-time gestational age estimation after the first trimester.

## Introduction

The fetal brain undergoes dramatic morphological and architectural changes within a short timeframe. Accurate understanding of key milestones in fetal brain maturation is critical for assessing range of normal development and long-term cognitive outcomes^1^. Previous studies have established an approximate spatiotemporal timetable of healthy fetal brain development, outlining the progressive gyrification of the cerebral cortex starting in the mid-second trimester^2–5^. Depending on severity, deviations from this pattern have been associated with developmental delays, psychomotor retardation, and failure to thrive^6^. The link between gestational age and cortical folding lays the foundation for neuroimaging-derived age predictions.

A growing body of neuroscience research has managed to leverage multiple imaging modalities to accurately predict the “brain age” of individuals using machine learning^7–9^. These algorithms learn the relationship between neuroimaging features and corresponding ages, after which they are tested on unseen data. Assuming model accuracy, discrepancies between estimated brain age and actual chronological age might suggest developmental brain pathology^10^. However, most studies to date have focused primarily on degenerative diseases and trauma in adults^11–14^. Fetal brain-based age estimation remains a major research gap and holds profound implications for obstetric prenatal care, delivery planning, and postnatal outcomes^9,15,16^.

The current method of choice for evaluating fetal brain maturity involves initial ultrasonography (US) of the cerebral cortex^17^. However, US can be severely limited by technical challenges and patient factors including maternal obesity, suboptimal fetal positioning, and oligohydramnios^18^. In addition, US-guided gestational dating in the second and third trimesters can err by up to 2 and 4 weeks, respectively^19^. In utero MRI has emerged as an important adjunct to US, offering detailed resolution of cortical gyration and myelination^20^. Nevertheless, rapid and ongoing neurodevelopmental changes, low signal-to-noise ratio, tissue contrast, and geometric distortions of small fetal brain embedded within the maternal structures pose obstacles to fetal neuroimaging. Fetal motion is also random, spontaneous, and possible in all planes, rendering even fast single-shot sequences challenging^21,22^. Furthermore, fetal brain MRI protocols, imaging platforms, and operator experience differ widely across institutions, leading to inconsistency in image quality and interpretation^23^.

Deep learning algorithms offer a powerful means to solve complex tasks such as fetal age estimation from highly variable imaging data^12,24–26^. Recent efforts have employed deep learning techniques on fetal brain MRI to infer gestational age, achieving moderate to high prediction accuracies^27,28^. However, these studies do not demonstrate a large or diverse enough sample to claim sufficient robustness or scalability^28,29^. The performance of some of these convolutional neural networks (CNN) also depends on manual brain segmentation, which can be time-intensive, poorly generalizable, and sensitive to artifacts, particularly in fetal imaging^30^. To address these problems, we proposed a self-attention framework to improve brain localization and the use of input images in multiple planes to maximize image diversity. We developed and tested several fully automated CNN architectures on a large heterogeneous single-center fetal MRI dataset. Finally, we tested the accuracy of age prediction when applied to data from several other centers of excellence in fetal imaging.

## Results

### Stanford cohort

A total of 741 T2-weighted MRI scans corresponding to unique patients (median gestational age 30.6 weeks, range 19–39 weeks) were included. Coefficient of determination (R^2^) and mean absolute error (MAE) for each model architecture tested are presented in Table 1. For each MRI plane, diminishing performance was seen with more than 3 input slices. Between the two age prediction approaches, averaging the outputs from the global branch and attention-guided local branch generated higher R^2^ scores and smaller MAE compared with predictions based on global images alone. The highest performing single-plane model was the attention-guided, 3-slice, coronal-view model with an R^2^ of 0.924 and corresponding MAE of 7.9 days.

**Table 1:**
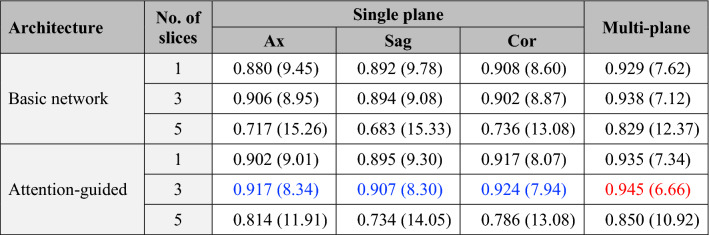
R^2^ score and mean absolute error performance across model architectures.

R^2^ scores and corresponding MAE (days) are shown for each model architecture. ResNet-50 was used as the backbone. The Basic Network analyzes the entire image as input without attention masking. For each column, the best performance based on R^2^ score and mean absolute error is colored in blue. The highest performing architecture across all tested permutations is in red.

**Figure 1:**
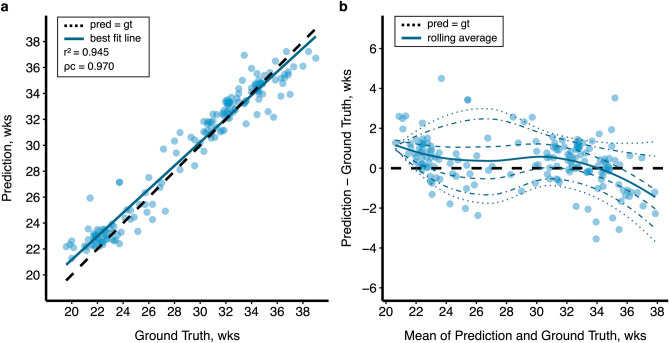
Regression performance of an attention-guided multi-plane ResNet-50 model. Model performance of the highest-scoring architecture visualized above. (**a**) Correlation between predicted brain age and ground truth (R^2^ = 0.945) is represented by the line of best fit (blue). The dashed line is the ideal regression, where prediction equals true age. (**b**) Differences between predictions and ground truth are shown on the modified Bland–Altman plot. Corresponding 5%, 10th, 25th, 50th, 75th, 90th, and 95th quantile curves based on local piecewise regression analysis are drawn.

Integrating information from the three planes achieved a notable improvement in model regression performance. A visualization of model regression performance is shown in Fig. 1. The concatenated multi-plane network produced the most accurate gestational age predictions out of all models tested, with the 3-slice architecture slightly outperforming the 1-slice model (R^2^ = 0.945 vs. 0.935; MAE = 6.7 vs. 7.3 days). The agreement between prediction and ground truth for this model was substantial based on Lin’s concordance correlation coefficient (ρc = 0.970; 95% CI 0.961–0.978). The modified Bland–Altman plot shows slight age overestimation up to about 34 weeks, after which the model progressively underestimates gestational age across quantile curves.

### External sites

The attention-guided, multi-plane ResNet-50 models trained on Stanford data were tested on external data obtained from four centers of excellence: Children’s Hospital of Los Angeles (CHLA), Cincinnati Children’s Hospital Medical Center (CCHMC), St. Joseph Hospital and Medical Center (SJH), and Tepecik Training and Research Hospital (TTRH). Without transfer learning, the 1-slice and 3-slice models achieved R^2^ of 0.690–0.861 and 0.523–0.857 and MAE of 9.2–16.0 days and 10.3–21.0 days, respectively. As shown in Table 2, both models demonstrated notable improvement after fine-tuning (ΔMAE = − 0.7 to − 4.1 days and − 0.5 to − 4.6 days). Combining all datasets, the 1-slice model achieved higher Lin’s concordance correlation coefficient than the 3-slice model, but the difference was not significant (ρc = 0.920 [0.903–0934], vs. 0.895 [0.874–0.913]). The most generalizable models were the fine-tuned 1-slice model for CHLA, SJH, and TTRH and 3-slice model for CCHMC, with R^2^ of 0.81–0.90, MAE of 8.4–12.9 days, and moderate ρc of 0.90–0.94.

**Table 2:**
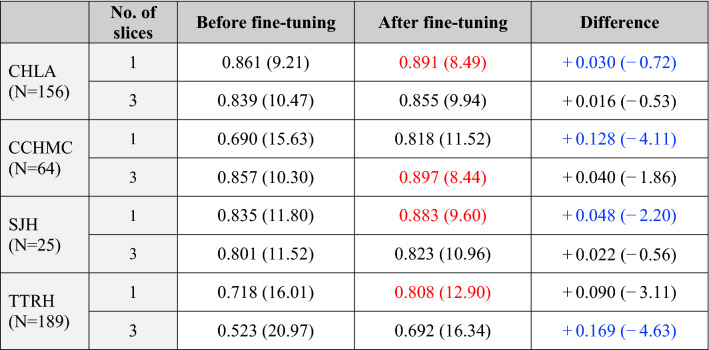
External validation of attention-guided, multi-plane, 1-slice and 3-slice models.

R^2^ scores and corresponding MAE (days) are shown before and after fine-tuning on data from other institutions. This external validation uses the highest-scoring model architecture (attention-guided multi-plane) based on the Stanford dataset. 20% of each external dataset was used for fine-tuning and the other 80% for testing model performance and generalizability. The largest improvements in R^2^ and MAE are shown in blue for each dataset. The most generalizable architecture for each dataset is in red.

## Discussion

In this study, we present an end-to-end, automated deep learning architecture that accurately predicts gestational age from developmentally normal fetal brain MRI. Our highest-scoring model performed at R^2^ of 0.945 on the Stanford test set, comparable or superior to published child, adolescent, and adult brain age prediction CNNs^8,10,24^. Previous works in fetal brain-based age analysis using MRI have primarily been limited to the development of spatiotemporal atlases for comparative age estimation and morphological segmentation^31–33^. Importantly, these methods help characterize fetal brain development and normal variability within the population^9^. However, most studies are restricted to a relatively small database, narrow age range, or isolated anatomical region (e.g., cortex, ventricles, hippocampus)^31,34–36^. These limitations reduce the generalizability of age-specific templates and reveal an important gap in our understanding of normal fetal brain maturation.

Variability in imaging quality presents another significant challenge for assessing fetal development. Challenges to interpretation include the rapidly changing neurological features in utero as well as the technical complexity of imaging^17,21^. Fetal MRI is notoriously complicated by the low signal from small fetal organs and relatively noisy background due to spontaneous fetal motion and maternal soft tissues (see Supplementary Fig. S1)^37,38^. One study showed that a deep learning segmentation model achieves high Dice overlap scores (96.5%) on clean datasets but low performance on images with motion artifact or abnormal fetal orientation (78.8%)^30^. This discrepancy highlights the importance of leveraging heterogeneous datasets to train and fine-tune deep learning networks. Accordingly, we reviewed all normal fetal MRIs at Stanford from 2004 to 2017 and excluded images only if severe imaging artifacts rendered them nondiagnostic. Our database of 741 images thereby enabled us to capture broad within-institution imaging variability and outnumbers datasets previously used to develop spatiotemporal atlases^9,31–33,39^.

More recent deep learning methods have utilized attention guidance in conjunction with object segmentation to improve noise resiliency^40,41^. Shi et al.^28^ built an attention-based deep residual network based on 659 pre-segmented fetal brains, achieving R^2^ of 0.92 and MAE of 0.77 weeks. Their use of attention activation maps emphasized global and regional features, such as cerebral volume and sulcal contours, within pre-processed segmentations to enhance prediction accuracy. However, this staged deep learning approach relies on the careful delineation of fetal brain masks, a time-intensive process that the authors report taking 30–40 min per sample. Since age regression depends on accurate object masking, external generalizability may be limited, as any fine-tuning would require manual segmentation by a trained researcher with domain knowledge. In contrast, we employ the attention mechanism to automatically focus on the fetal brain itself, enabling a higher signal-to-noise ratio by excluding unrelated features such as the maternal organs and other fetal body parts and reducing non-uniform MR intensity. Furthermore, both attention-guided masking and age regression are trained simultaneously and recursively, obviating the need for extensive pre-processing and fine-tuning. Our best-performing model was thereby computationally efficient and scalable, completing its regression task within 5 min at a GPU level.

The real-world utility of any deep learning model largely depends on its generalization performance. For fetal MRI in particular, standard imaging protocols, quality of imaging, sequences used, and operator experience differ widely across institutions^23^. Performance losses incurred when transferring models from one institution to another has become a major concern in the machine learning field. In this study, we test multi-center generalizability of our automated deep learning network using a large external database spanning four centers of excellence, two countries, and a wide array of imaging platforms, scanner hardware, and acquisition parameters (Table 3). There were visible differences in image appearance when comparing datasets across different sites due to factors such as resolution, contrast, and signal-to-noise ratio (see Supplementary Fig. S2). Accordingly, our Stanford-trained multi-plane models yielded varying degrees of performance reduction on the external datasets. However, fine-tuning the model with just 20% of the external data enabled the network to adapt to the new cohort, highlighting its potential applicability across institutions and imaging platforms. Meaningful improvements in R^2^ score, MAE, and age concordance were achieved across institutions after fine-tuning and may continue to be observed using larger validation datasets.

**Table 3:** MRI datasets and acquisition parameters by institution.

	Institution								
	Stanford	CHLA		CCHMC		SJH		TTRH	
No. of subjects	741	156		64		25		189	
Median GA (range), wks	30.6 (19–39)	30.6 (20–40)		24.6 (16–39)		28.7 (19–36)		26.1 (18–40)	
Field strength	1.5 T, 3 T	1.5 T, 3 T		1.5 T		1.5 T, 3 T		1.5 T	
Manufacturer and Scanner	GE Discovery 750 W, Optima 450 W, Signa HDxt & Excite	Philips Ingenia & Achieva		GE Signa HDxt Philips Ingenia		GE Signa HDxt & Excite		Siemens Magnetom Aero & Avanto	
Sequence	ssFSE	ssTSE		ssFSE, ssTSE, bTFE, FIESTA		ssFSE, FIESTA		HASTE, TRUFI	
Repetition time, ms	600–6,000	750–2625	12,500–15,000	3–5	4000	4.6–4.9	1,300–2,300	3.6–5.0	1200–1700
Echo time, ms	67–420	70–120	90–120	1.5–2.3	80–120	1.9–2.1	78–93	1.4–2.0	104–198
Flip angle	90°	90°		75°–110°		75°, 90°		62°–180°	
Field of view, mm	180 × 180–440 × 440	160 × 160–450 × 450		240 × 240–380 × 380		240 × 240–340 × 340		129 × 187–380 × 380	
In-plane resolution	0.35 × 0.35–1.57 × 1.57	0.48 × 0.48–1.28 × 1.28		0.55 × 0.55–1.37 × 1.37		0.46 × 0.46–0.67 × 0.67		0.37 × 0.37–1.66 × 1.66	
Median no. of slices (range)	23 (7–48)	44 (20–100)		20 (5–53)		22 (14–47)		26 (10–84)	
Median slice thickness (range), mm	4 (2–5)	3 (2.5–5.5)		4 (3–6)		4 (4–5)		4 (3–5)	

*ssFSE* single-shot fast spin-echo, *ssTSE* single-shot turbo spin-echo, *bTFE* balanced turbo field echo, *FIESTA* fast imaging employing steady state acquisition, *HASTE* half-Fourier acquisition single-shot turbo spin-echo, *TRUFI* true fast imaging with steady-state free precession.

Fetal MRI not only offers insight into prenatal development, but can also guide laboratory work-up, therapeutic interventions, counseling, and delivery planning^23^. At present, the reported date of last menstrual period and first-trimester US measurements are “gold standard” methods for determining gestational age^19^. However, inaccurate recall of the last menstrual period, confounding factors (e.g., irregular spotting or ovulation), and US variability in the second and third trimesters have propelled the need for alternative gestational dating approaches^42^. In our study, fetal brain MRI scans interpreted as normal based on expert consensus were used to develop a convolutional neural network that was highly predictive of gestational age, offering a potential solution for age estimation in the second half of gestation. Our end-to-end approach to assessing the fetal brain also obviates the need for manual feature engineering or segmentation, enabling real-time interpretation. Moving forward, this model may serve as a backbone for evaluating gestational age as well as deviations from normal development, such as underdevelopment, malformation, and other congenital diseases^6,9^. Furthermore, emerging deep learning techniques in image reconstruction^43^ offer promise for developing population-based spatiotemporal atlases to better characterize age-based fetal neuroanatomy.

There are several limitations to this study. As a 2D CNN, age predictions are made based on single-slice inputs, potentially limiting the information available to the network. A 3D CNN incorporating multi-slice imaging features may improve model performance but would require a much larger dataset and risk greater background noise. Our approach to enhance regression accuracy involves Gaussian weighting of the attention heatmap, optimized for images centered on the fetal brain. Extreme position and size variability thereby reduces the accuracy of attention-guided mask inference but not necessarily regression performance as shown in Supplementary Fig. S3. This may be explained by the inclusion of both local and global branches, incorporating semantic features from the emphasized subregion as well as the entire image, respectively. A drawback of this approach is the inclusion of unwanted background noise when the localization procedure performs optimally.

Notably, beyond 34 weeks, our model appears to underestimate gestational age. This trend can be partially attributed to dataset imbalance with few fetal MRI performed in the late third trimester, biasing predictions toward younger gestational ages. US and MR imaging studies also indicate that peak gyrification occurs between weeks 29–35 and that most of the primary and secondary sulci along with all notable gyri have formed by weeks 34–37^4,18,44^. A decreasing gyrification rate approaching full term may also skew age estimates, as fetal brains appear more homogenous as they near maturity. Future work can extend the training set to include fetal MRI at age extremes and explore emerging methods such as feature distribution smoothing for imbalanced data with continuous labels^45^. In terms of generalizability, our model may also benefit from the inclusion of external data in the original training set to reduce over-fitting. Finally, a machine learning model is only as reliable as the quality of its input data. Long-term clinical and developmental outcomes for our cohort are unavailable, so scans used to train and test our model are only “normal” from a neuroanatomical perspective.

## Conclusion

Deep learning has emerged as a powerful approach for interpreting complex image features. We present an attention-guided, multi-view deep learning network that analyzes MRI-based features of the normally developing fetal brain to accurately predict gestational age. We further demonstrate model performance on external sites and the utility of fine-tuning the model for enhanced generalizability. This study identifies opportunities for imaging-driven analytics of in utero human neural development with potential to enhance diagnostic precision in the second and third trimesters.

## Materials and methods

### Stanford data collection and cohort description

We retrospectively reviewed all 1927 fetal brain MRIs performed at Stanford Lucile Packard Children’s Hospital from 2004 to 2017, as described in Supplementary Table S1. 1.5 T and 3 T MRI data were acquired with an 8-channel head coil on Signa HDxt, Signa EXCITE, Optima MR450W, and Discovery MR750W scanners (GE Healthcare). 572 images containing cerebral malformations, ventriculomegaly, or other acquired or congenital brain lesions were excluded. 422 nondiagnostic images with severe motion artifacts or noise preventing adequate interpretation were also omitted. In total, we compiled a database of 933 fetal brain MRIs, interpreted as developmentally normal by expert pediatric neuroradiologists. MRI interpretations were based on visual features and biometry measurements such as brain biparietal diameter and skull occipitofrontal diameter. 741 studies had single-shot fast spin-echo T2-weighted sequences in all three planes (axial, coronal, and sagittal). The single-shot images, originally in DICOM File Format, were compressed to JPG files for visualization. The image slices near the middle of the sequence were pre-processed and augmented as the input. Slices were randomly cropped to 224 × 224 and normalized using sample mean and standard deviation. These data were randomly split into training (70%), validation (10%), and test (20%) sets for model input.

This study was approved by Stanford University’s Institutional Review Board (IRB). Data collection and analysis were performed in accordance with relevant guidelines and regulations. Written informed consent was obtained from all pregnant women or authorized representatives for imaging of fetuses prior to delivery (IRB protocol #42137).

### Model structure

**Figure 2:**
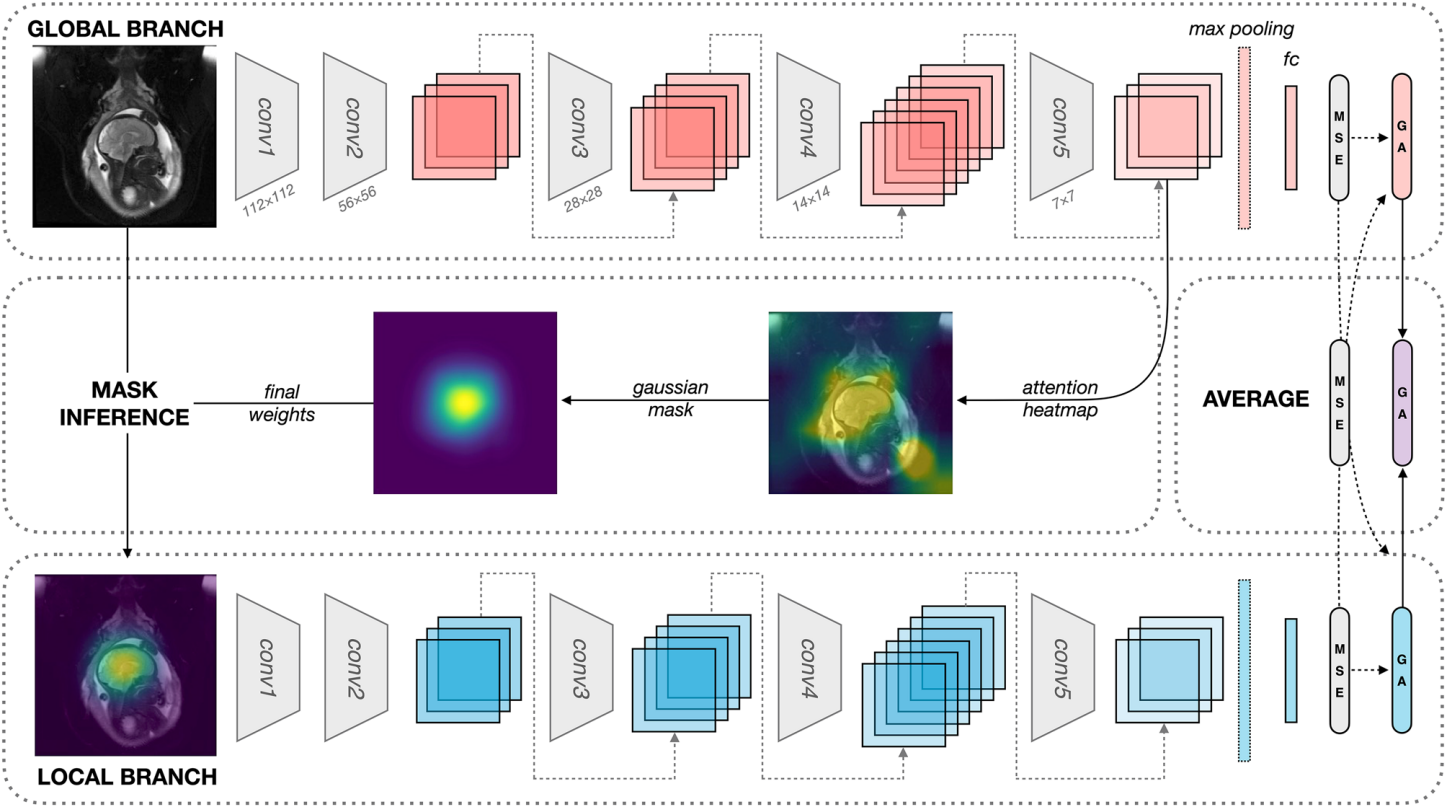
ResNet-50 architecture for brain age regression with attention-guided mask inference. A single sagittal image with dimensions 224 × 224 is shown as an input to the global branch. Architectures incorporating multiple slices and planes are not displayed. The input of the local branch is a weighted image isolating the region of interest automatically generated from attention-guided mask inference. Global and local branches contain five convolutional layers (conv1 to conv5), each consisting of 3–6 building blocks (boxes) with a convolution, batch normalization, and rectified linear unit (ReLU), streamlined by shortcut connections (gray dotted arrows). Output sizes are denoted by *k* × *k*. Feature maps from both branches enter a max pooling layer and are subsequently fed to a fully connected layer (fc). The MSE for each branch and the total loss are minimized via gradient descent (black dotted arrows), simultaneously tuning model weights for both local and global branches via backpropagation. Age predictions (GA) are generated from each branch and averaged to produce the final age estimation.

The model architecture consists of two parallel branches, the global and local branches, as shown in Fig. 2. Both the global and local branches consist of deep residual neural networks that are optimized to predict gestational age based on fetal MRI. ResNet-50, a CNN pre-trained on more than a million images from the 2012 ImageNet database, was used as the backbone deep neural network for age regression^46^. For each stack of input image slices, we assumed the middle slice to contain the largest fetal brain area. We then tested the effect of the number of image slice inputs on model performance (e.g., 1, 3, 5), incorporating additional slices immediately adjacent to the middle slice. The first convolutional layer of the ResNet-50 model was parameterized to accommodate different numbers of image slices with their corresponding input channels. Pretrained model weights were then applied to subsequent layers of the network. Given input image(s) *X*, the global branch is first trained using the entire or ‘global’ *X*. Then, the region of interest is masked using an attention mechanism with Gaussian weighting and trained for age regression on the local branch. Learned features from both branches simultaneously optimize final age prediction. Independent models were trained on axial, coronal, and sagittal images to study the unique semantic features from different planes.

We compared two approaches for predicting gestational age *y*_*pred*_: global branch predictions (i.e., entire image) without the attention-guided local branch, versus averaged age predictions from both the global branch and local branch (i.e., masked region of interest). The true gestational age *y*_*true*_ or ‘ground truth’ was determined via the standard-of-care approach of estimating the date of delivery based on an early obstetric ultrasound in the first trimester^19^. Gestational ages at time of US were recorded directly from the reports, and differences in MRI and US dates were added to obtain *y*_*true*_ for each patient. In the training phase, the model is optimized by stochastic gradient descent with backpropagation to minimize the mean squared error (MSE) loss between true and predicted ages$y_{true} - y_{pred\;2}^{2}$ ^47^.

### Attention-guided mask inference

Computational analysis of fetal MR imaging is extremely challenging due to the random position and rotation of fetal brains across patients. Additionally, noise unrelated to the fetus (such as the maternal placenta and organs) may negatively affect predictive performance. These considerations motivated the use of attention-guided mask inference, which provides spatially variant maps that highlight regions of interest and contribute to accurate object recognition^48^.

As previously described in Guan et al.^49^ and Zhou et al.^50^, the attention heatmap is extracted from the last convolutional layer in the global branch. Given an initial input image *X* representing the whole image slice, $f_{k} \left( {x,y} \right)$ represents the activation of spatial location $\left( {x,y} \right)$ in the *k*th channel of the output of the last convolutional layer, where $k \in \left\{ {1, \ldots ,\left. K \right\}} \right.$ and *K* is the total number of feature map channels ($K = 512$ in ResNet-18, $K = 2048$ in ResNet-50). The attention heatmap values $H_{g}$ are computed by maximizing activation values across channels:

$$H_{g} \left( {x,y} \right) = \max \left( {\left| {f_{k} \left( {x,y} \right)} \right|} \right), \quad k \in \left\{ {1, \ldots ,\left. K \right\}} \right.$$

**Figure 3:**
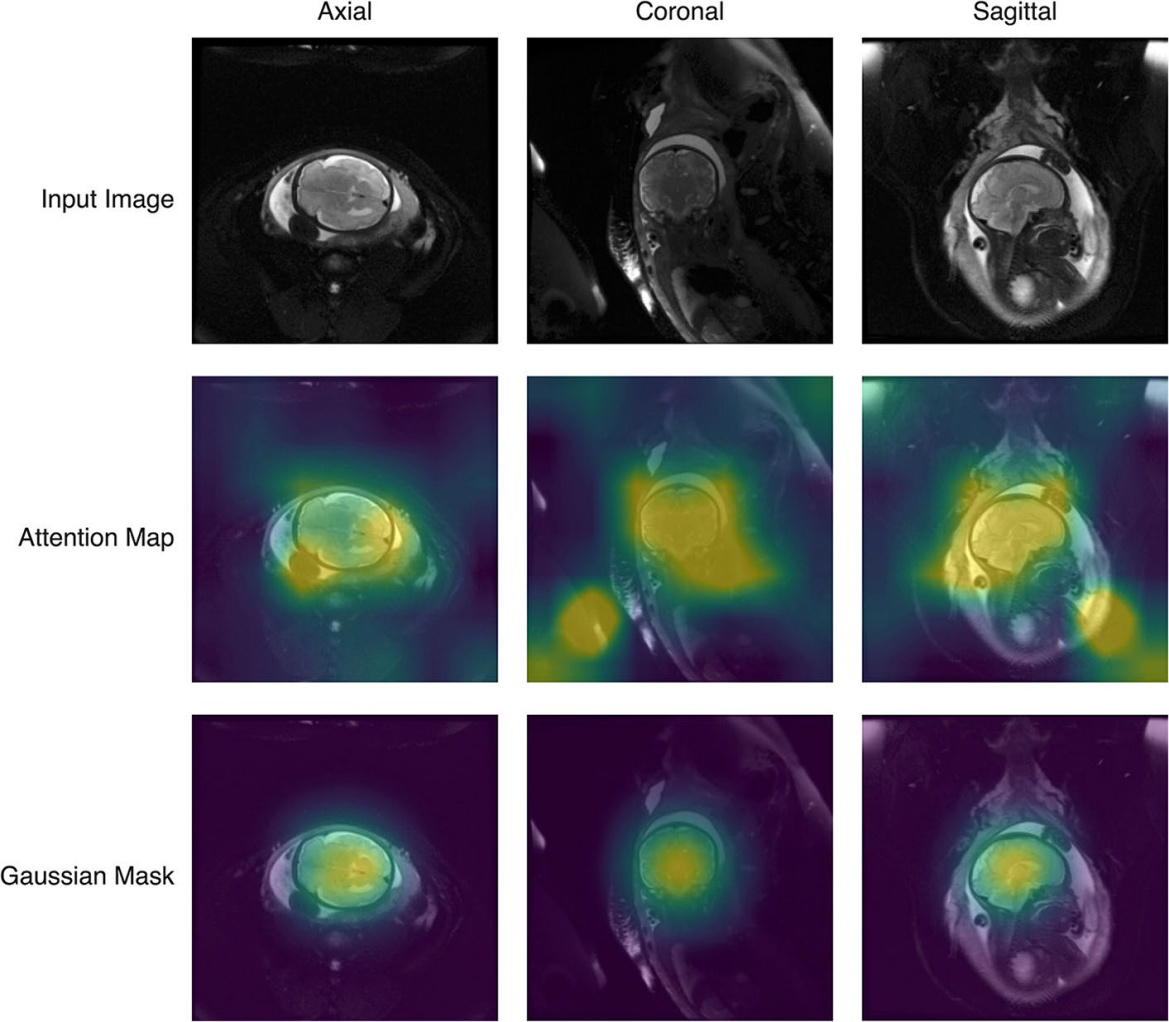
Examples of heatmap generation and region of interest mask inference. Top: Global input images show the entire view of a maternal womb captured on MRI in all three planes. Middle: Corresponding heatmaps derived from the last convolutional layer identify high-value areas for attention-based learning. Increasing activation values correspond to the color spectrum from violet to yellow. Bottom: Application of a 2D Gaussian mask generates a re-weighted heatmap highlighting the region of interest.

After up-sampling $H_{g}$ to match the resolution of the input images, we apply the truncated ReLU activation function to normalize the heatmap $H_{g}$ to the data range of [0, 1], where larger values represent increasing probability of detecting fetal brain tissue. High-value areas are subsequently given more attention by the prediction model. Furthermore, with the prior knowledge that the fetal brain usually localizes in the center of the image, we multiply a 2D Gaussian mask to re-weight the heatmap. Thereafter, the heatmaps highlighting the region of interest (i.e., fetal brain) are generated. Examples of heatmaps are shown in Fig. 3.

Heatmap weights are multiplied with the input image to obtain a masked region of the fetal brain, suppressing background noise in the original scan. The re-weighted image is then inputted to the local branch for age prediction based on regional features. Since we automatically extract the heatmap from the global branch and the normalization operations are differentiable, the entire model framework can be trained end to end for adaptive attention map weighting and brain age estimation.

### Multi-plane learning approach

A multi-plane learning approach was employed to capitalize on complementary information contained in different MRI dimensions. Separately from the single-plane architectures, we trained a multi-plane model by minimizing the total MSE loss involving axial, coronal, and sagittal planes. Network weights are thereby optimized based on features from all MRI views simultaneously. After convergence, prediction outputs from each plane are then averaged for a final estimation of gestational age.

### Training and evaluation

All network architectures were implemented with the PyTorch framework^51^. We trained the models using the Adam Optimizer with a learning rate of 1 × 10^−4^ and a batch size of 50 for 2000 iterations. The training session was conducted on a NVIDIA TITAN Xp GPU. High scoring models were defined as those with strong correlation and concordance between true gestational age and predicted gestational age. Correlative strength was evaluated for all models trained and tested on Stanford fetal imaging data by the R^2^ and MAE. Concordance between predicted and true gestational ages was determined using Lin’s concordance correlation coefficient, with strength of agreement assessed by McBride’s criteria as follows: poor, < 0.90; moderate, 0.90–0.95; substantial, 0.95–0.99; almost perfect > 0.99^52,53^. Statistical results were visually confirmed by local piecewise regression analysis using a window size of 15 points, 95% overlap between windows, and Gaussian smoothing^54^.

### Validation with external sites

External MRI data were obtained from four additional centers of excellence: Children’s Hospital of Los Angeles, Cincinnati Children’s Hospital Medical Center, St. Joseph Hospital and Medical Center, and Tepecik Training and Research Hospital in İzmir, Turkey. MR imaging across sites varied widely in terms of scanning platform, sequence types, and technical settings, as shown in Table 3. To test generalizability, the attention-guided multi-plane model (i.e., highest-scoring network tested on Stanford data) was used. The 1-slice and 3-slice architectures were compared across external institutions. After deploying the same data curation methods used for Stanford data, the external datasets consisted of 156, 64, 25, and 189 fetal MRI samples for CHLA, CCH, SJH, and TTRH, respectively (Supplementary Fig. S2). The Stanford-trained model was first tested directly on these unseen external samples without any transfer learning. We then fine-tuned the model with 20% of each dataset using the Adam optimizer with a learning rate of 1 × 10^−5^ and a batch size of 5. For SJH, we used a learning rate of 1 × 10^−6^ as only 5 data samples were available for fine-tuning. We employed early stopping at 5 epochs to avoid overfitting. Performance with and without fine-tuning on the remaining 80% of each dataset was compared.

## Supplementary Information

Supplementary Information.

## Data Availability

Deidentified images used in model training and testing are made available at the Stanford Digital Repository (https://purl.stanford.edu/sf714wg0636). All requests for raw data and related materials will be reviewed by the Office of the General Counsel at Stanford University to verify whether the request is subject to any intellectual property or confidentiality obligations. Restrictions generally apply to the public availability of the data due to patient agreements and privacy concerns. Any data and materials that can be shared will be transferred securely via a formal data sharing agreement.

## Code availability

Source code and tutorial will be made available to reviewers upon request and deposited in a DOI-minting repository upon acceptance for publication. We used the publicly available ResNet-50 network as the backbone architecture for our deep learning models, available at https://github.com/pytorch/vision/tree/master/torchvision/models.
