# Supplementary material for: Attention-guided deep learning for gestational age prediction using fetal brain MRI
Source: Sci Rep. 2022 Jan 26;12:1408. doi: 10.1038/s41598-022-05468-5 (PMC8791965; doi:10.1038/s41598-022-05468-5)
Supplement: Supplementary file 1 — Supplementary Information. [file 41598_2022_5468_MOESM1_ESM.pdf]

# Supplementary Material

Shen L, Zheng J, Lee EH, Shpanskaya K, McKenna ES, Atluri MH, Plasto D, Mitchell C, Lai LM, Guimaraes CV, Dahmoush H, Chueh J, Halabi SS, Pauly JM, Xing L, Lu Q, Oztekin O, Kline-Fath BM, Yeom KW. Attention-Guided Deep Learning for Gestational Age Prediction using Fetal Brain MRI. *Sci Rep*. Published online December 2021.

**Table of Contents**


| | |
|---|---|
| Supplemental Figure S1. Common artifacts and variations in multi-plane fetal brain imaging | 2 |
| Supplementary Figure S2. Example of cropped multi-plane fetal images from external institutions | 3 |
| Supplementary Figure S3. Variability in attention masking based on fetal brain position, size, and background noise | 4 |
| Supplementary Table S1. Overview of Stanford Fetal Cohort and MRI Indications | 5 |

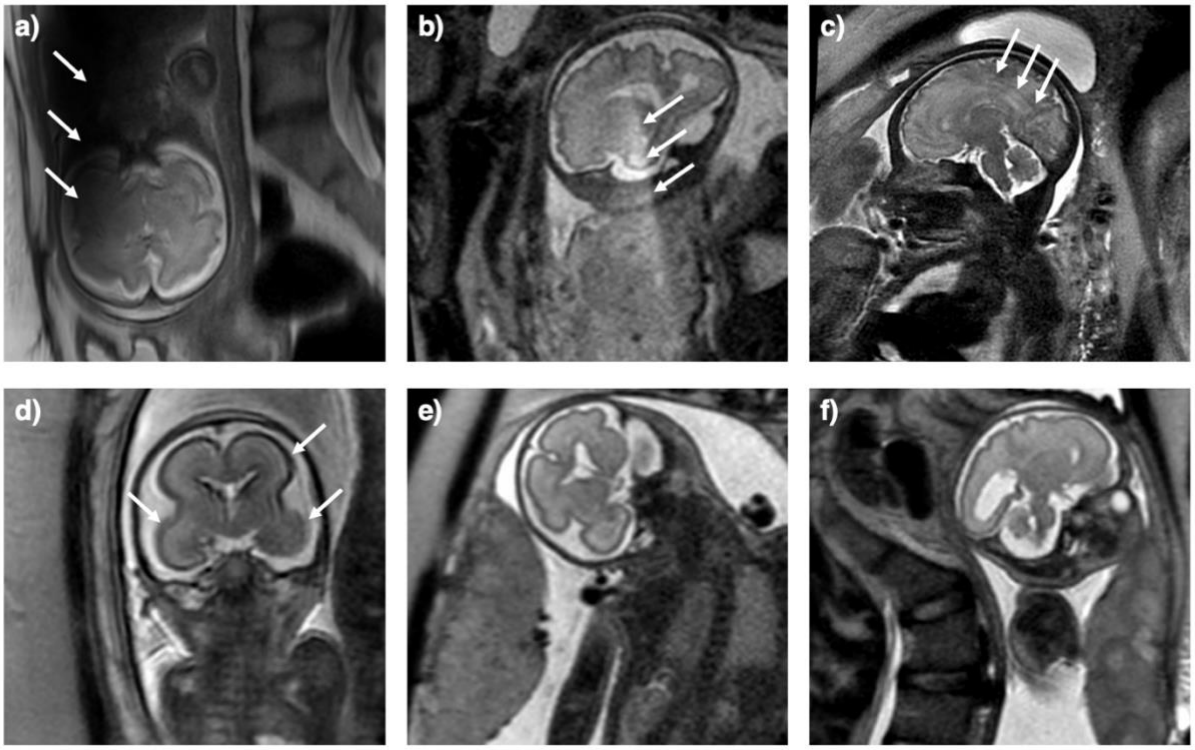


**Supplementary Figure S1. Common artifacts and variations in multi-plane fetal brain imaging. (a)** Signal drop-out ipsilateral to the fetal brain and body (arrows) is seen stemming from dielectric effect artifact. **(b)** Linear high signal (arrows) is seen representing wrap-around or aliasing artifact. **(c)** Multiple curvilinear signal (arrows) could mimic cortical malformation, but most likely represents motion artifacts, as evidenced by the regions below the brain. **(d)** Fetal motion has resulted in image blurring (arrows) at brain cortical surface. **(e, f)** Oblique anatomic imaging commonly occurred due to variations in fetal position at image.

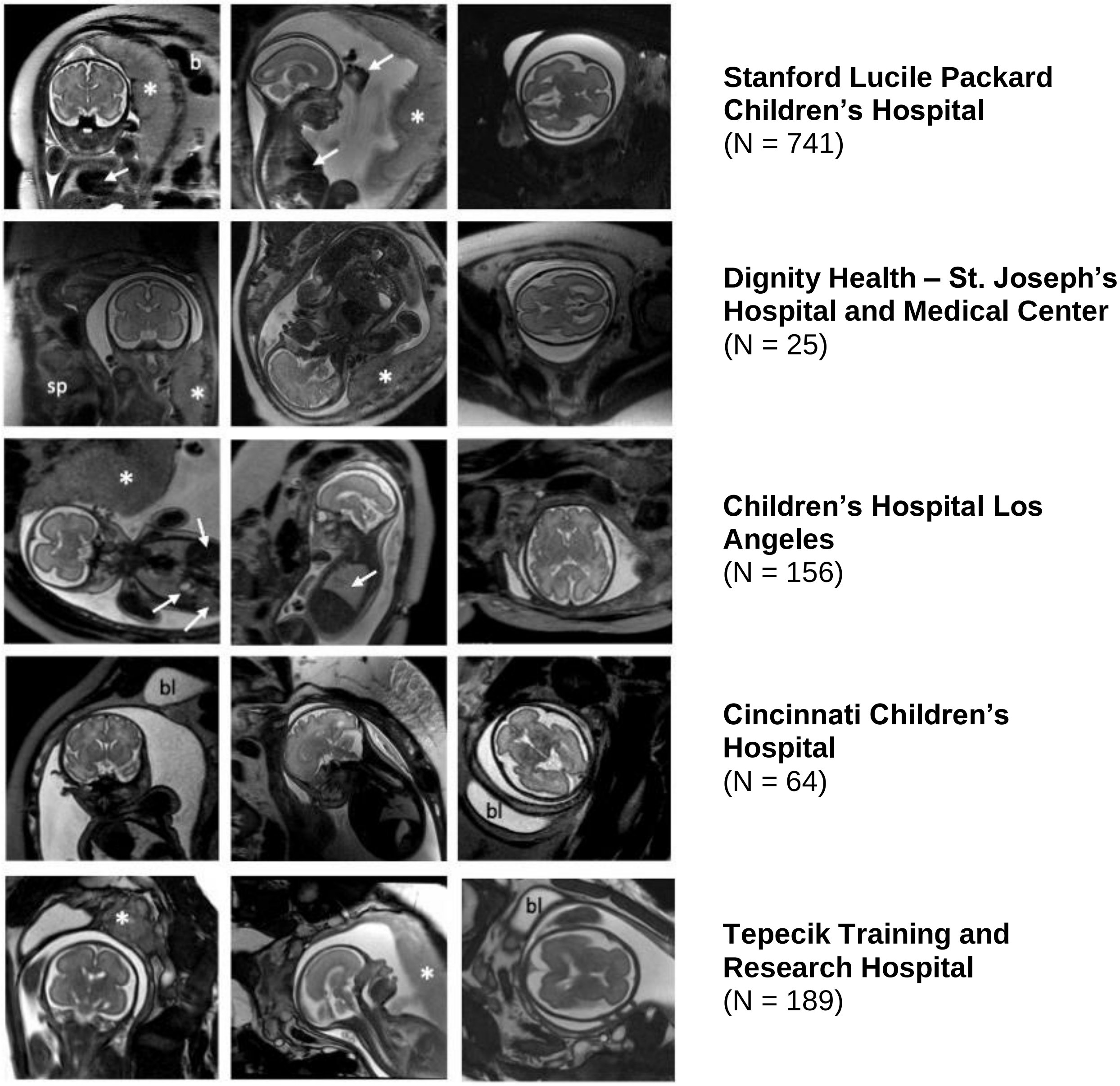


**Supplementary Figure S2. Example of cropped multi-plane fetal images from external institutions.** From top to bottom: Stanford Lucile Packard Children's Hospital, St. Joseph's Hospital and Medical Center, Children's Hospital Los Angeles, Cincinnati Children's Hospital, and Tepecik Training and Research Hospital. In addition to maternal structures, such as bladder (bl), bowel (b), and spine (sp), placenta (*), non-neural fetal structures (arrows), such as lung, kidney, heart, liver, gallbladder, and limbs were often included in the image field of view.  Fetus position, size, and background noise varied widely within and across datasets.

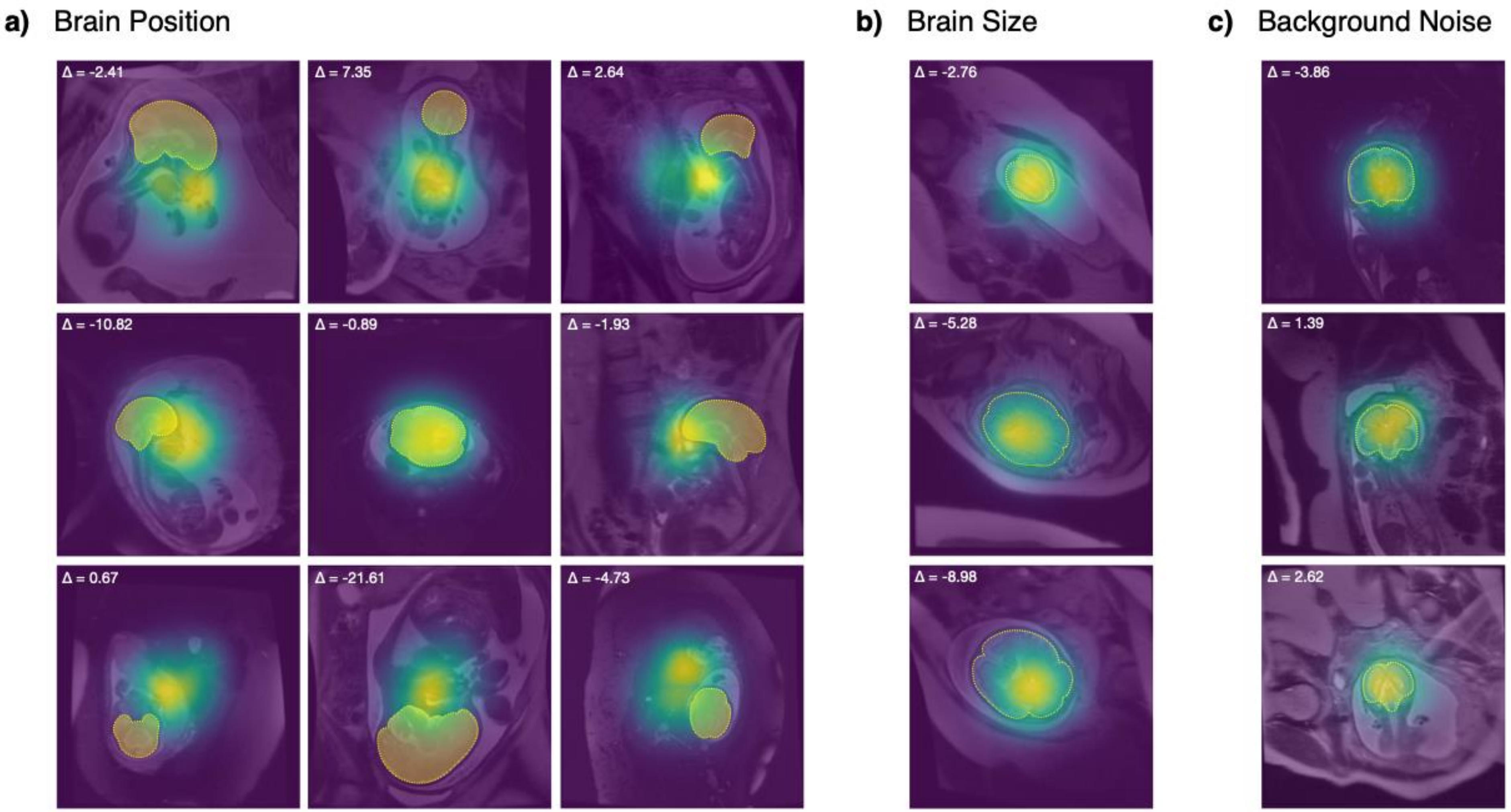


**Supplementary Figure S3. Variability in attention masking based on (a) fetal brain position, (b) size, and (c) background noise.** The difference between estimated and true gestational ages in days, as predicted by the corresponding single-plane 1-slice model, is displayed in the upper left corner of each image (*Δ prediction – ground truth*). As visualized, the attention mechanism performs variably across different degrees of non-uniformity, showing the most resilience to background noise and the least to positional variation. Inclusion of the global branch (i.e., entire image) promotes performance stability by guaranteeing semantic feature extraction from the fetal brain despite imprecise localization.

**Supplementary Table S1. Overview of Stanford Fetal Cohort (N=741) and MRI Indications**

| Top indications for MRI, by category[a] | N (%)[b] | | N (%) |
|---|---|---|---|
| ***Pregnancy Conditions*** | **43 (5.8)** | ***Cardiovascular*** | **27 (3.6)** |
| Oligo- or anhydramnios | 15 | Congenital heart defect (TOF, VSD, ASD, etc.) | 14 |
| Polyhydramnios | 14 | Dextrocardia | 2 |
| IUGR | 4 | Ectopia cordis | 2 |
| Other | 10 | Echogenic intracardiac focus | 2 |
| | | Other | 7 |
| ***Family History*** | **13 (1.8)** | | |
| Hereditary hemorrhagic telangiectasia | 2 | ***Gastrointestinal*** | **140 (18.9)** |
| Joubert syndrome | 2 | Bowel dilation | 44 |
| Polymicrogyria | 2 | Abdominal mass or cyst | 29 |
| Other | 7 | Increased bowel echogenicity | 11 |
| | | Small stomach | 10 |
| ***Central Nervous System*** | **134 (18.1)** | Ascites | 8 |
| Absence of cavum septum pellucidum | 29 | Other | 38 |
| Enlarged cisterna magna | 29 | | |
| Ventriculomegaly | 15 | ***Renal / Genitourinary*** | **119 (16.1)** |
| Cerebellar / cerebellar vermis hypoplasia | 8 | Multicystic dysplastic kidney | 16 |
| Dandy-Walker malformation | 6 | Pelvic / ovarian cyst | 16 |
| Other | 47 | Hydronephrosis / renal pelviectasis | 14 |
| | | Renal agenesis | 13 |
| ***Orofacial*** | **41 (5.5)** | Other | 60 |
| Cleft lip and/or palate | 20 | | |
| Microcephaly | 13 | ***Musculoskeletal*** | **106 (14.3)** |
| Micrognathia / retrognathia | 7 | Congenital diaphragmatic hernia | 40 |
| Other | 1 | Omphalocele / gastroschisis | 19 |
| | | Other | 47 |
| ***Pulmonary*** | **142 (19.2)** | | |
| Lung mass or cyst | 48 | ***Miscellaneous*** | **65 (8.8)** |
| CCAM / CPAM | 43 | Heterotaxy | 6 |
| Bronchopulmonary sequestration | 30 | Poor ultrasound visualization | 6 |
| Other | 21 | Other | 53 |

Abbreviation: IUGR, intrauterine growth restriction; CCAM, congenital cystic adenomatoid malformation; CPAM, congenital pulmonary airway malformation; EDD, estimated date of delivery; TOF, Tetralogy of Fallot; VSD, ventricular septal defect; ASD, atrial septal defect

[a] Indications above represent suspected pathologies by ultrasound but do not indicate final radiologist interpretation.

[b] Percent of unique patients with a given indication. Some patients had two or more indications for MRI.
